# Conditional survival of patients with primary bone lymphoma of the spine: how survival changes after initial diagnosis

**DOI:** 10.3389/fonc.2024.1356947

**Published:** 2024-05-01

**Authors:** Gang Zheng, Zhihao Yang, Hui Qian, Hua Huang, Zhiwei Gu

**Affiliations:** Department of Neurosurgery, Shaoxing Central Hospital, The Central Hospital of Shaoxing University, Shaoxing, Zhejiang, China

**Keywords:** primary bone lymphoma of the spine, conditional survival, nomogram, survival estimation, SEER primary bone lymphoma of the spine, SEER

## Abstract

**Background:**

The current survival prediction methodologies for primary bone lymphoma (PBL) of the spine are deficient. This study represents the inaugural utilization of conditional survival (CS) to assess the outcome of this disease. Moreover, our objective was to devise a CS-based nomogram for predicting overall survival (OS) in real-time for spinal PBL.

**Methods:**

Patients with PBL of the spine diagnosed between January 2000 and December 2015 were extracted from the Surveillance, Epidemiology, and End Results (SEER) database. The OS was determined through the Kaplan–Meier method. The CS characteristic of patients with spinal PBL was delineated, with the CS being estimated utilizing the formula: CS(α|β) = OS(α+β)/OS(β). CS(α|β) denotes the probability of additional α-year survivorship, assuming the patient has already survived β years after the time of observation. Three methods including univariate Cox regression, best subset regression (BSR) and the least absolute shrinkage and selection operator (LASSO) regression were used to identify predictors for CS-based nomogram construction.

**Results:**

Kaplan-Meier analysis was executed to determine the OS rate for these patients, revealing a survival rate of 68% and subsequently 63% at the 3-year and 5-year mark respectively. We then investigated the CS patterning exhibited by these patients and discovered the survival of PBL in the spine progressively improved with time. Meanwhile, through three different prognostic factor selection methods, we identified the best predicter subset including age, tumor histology, tumor stage, chemotherapy and marital status, for survival prediction model construction. Finally, we successfully established and validated a novel CS-based nomogram model for real-time and dynamic survival estimation. Moreover, we further designed a risk stratification system to facilitate the identification of high-risk patients.

**Conclusions:**

This is the first study to analyze the CS pattern of PBL of the spine. And we have also developed a CS-based nomogram that provide dynamic prognostic data in real-time, thereby aiding in the formulation of personalized treatment strategies in clinical practice.

## Introduction

Primary bone lymphoma (PBL) is a relatively uncommon type of neoplastic disease, accounting for approximately 1% of all lymphomas, 3-7% of extranodal lymphomas, and 7% of principal malignant bone tumors (1–3). And the estimated 5-year survival rate (OS) for PBL patients has been reported to fall within a range of approximately 55%-75% (4, 5). Non-Hodgkin lymphoma (NHL) constitutes the predominant category of PBLs with diffuse large B-cell lymphoma (DLBCL) accounting for more than 80% of all instances (2, 6). PBL can occur across all age groups, with a prevalent diagnostic age range of 45-60 years, and males are predominantly affected compared to females (2, 7, 8). PBL of the spine is an uncommon entity, typically arising as metastatic disease from additional sites (2). The clinical manifestations of spinal PBL are commonly non-specific, often resulting in a diagnostic delay, and usually necessitating a biopsy for a definitive diagnosis (5, 9–11). Owing to the scarcity of the PBL of the spine, there is a scarcity of comprehensive data on the optimal treatment strategy and prognosis. The prevailing modalities encompass chemotherapy or immunochemotherapy with or without radiotherapy; while surgical intervention are generally restricted (2). Furthermore, the late complications after chemotherapy in such patients are currently under scrutiny, including bone loss and the development of fragility fractures (12). And survival outcomes of these patients are yet to be investigated.

With the progression of medical technology, the substantial augmentation in the proportion of tumor patients with enduring survival heightens the necessity to comprehend the dynamic survival probability. Traditional evaluations intended for patient survival prediction, which are frequently from the time of diagnosis, may unable to present updated survival information for individuals who have survived several years post-diagnosis. As a varietal of extensively utilized statistical methodologies, conditional survival (CS) analysis offers an immediate recalculation of survival outcomes, delineating the evolution of survival probabilities across different time periods (13). And these dynamic and precise prognostic data can aid in optimizing the clinical management and follow-up strategies, facilitating enhanced physician-patient communication, and providing psychological support for these patients, which hold significant clinical value.

On the other hand, accurate prognosis evaluation necessitates integrating the clinicopathological characteristics and therapeutic status of patients. Despite traditional nomogram’s ability to personalize prognosis prediction via taking individual characteristics into account (14, 15), this methodology remains limited in its capacity to delineate the dynamic change in survival over time (16). Thus, incorporating CS within a nomogram holds potential for customized, timely and dynamic prognostic prediction.

Due to its rarity, our comprehension of PBL of the spine predominantly rests on case studies, with study in a substantial population remains deficient. Hence, in this current study, utilizing the Surveillance Epidemiology and End Results (SEER) database (17), we aimed to investigate survival outcomes of patents with PBL of the spine and to describe the CS profile of these patients at a population-based level. And we also developed and validated a novel CS-nomogram model for dynamic survival prognosis quantification for spinal PBL patients.

## Methods

### Patient population and clinical variables

We have conducted this retrospective study utilizing the SEER database, a well-regarded source for population-based cohort studies. Patients with PBL of the spine were extracted from the SEER database. Inclusion criteria were summarized as follows: (1) subjects diagnosed with PBL of the spine (International Classification of Disease for Oncology, Version 3 (ICD-O-3) histological codes 9590-9599, 9670-9699, 9700-9719, 9720-9729, and 9827); (2) confirmed between 2000 and 2015, (3) anatomical site code C41.2 and C41.4, (4) pathological diagnosis corroborated. The following exclusion criteria were employed: (1) pathological confirmation derived through postmortem examination; (2) deficiency in survival time, tumor stage, and surgical intervention data.

Given the constraints on the variables provided by the SEER database, we made every effort to incorporate those associated with PBL prognosis into this study. Moreover, psychological factors and socio-economic status are also considered to be independent prognostic factors of malignant tumors; hence, we also conducted analyses on variables such as marital status and patient household income. Finally, the following demographic, clinicopathological and therapeutic parameters were analyzed in our study: age, sex, race, marital status, rural-urban distribution, tumor site, tumor stage, tumor histology, surgery, radiation therapy, chemotherapy and patient household income. In our study, survival analysis based on the endpoint of OS, interpreted as the duration from the disease recognition to patient death, was implemented through the application of the Kaplan-Meier method.

### Conditional survival

Given that the CS analysis allows for the immediate recalculation of survival outcomes, the CS characteristic of patients with spinal PBL was further delineated, with the CS being estimated utilizing the formula (18):


CS(α|β) = OS(α+β)/OS(β)

CS(α|β) denotes the probability of additional α-year survivorship, assuming the patient has already survived β years after the time of observation. OS(α+β) and OS(β) signify the (α+β) and β year OS as estimated via the Kaplan-Meier method, respectively. For instance, CS(1|4) represents the probability of a 5-year CS wherein a patient who had survived 4 years after initial diagnosis and then lived for another 1 years.

### Statistical analysis

The data procured from the SEER database were categorized into training and validation groups proportionally at a ratio of 3:1. The chi-square test was employed to compare the categorical variables between the two groups. Subsequently, three methodologies including univariate Cox regression (P<0.05 as screening criteria), the least absolute shrinkage and selection operator (LASSO) regression (lambda.min is used as a screening criterion) and the best subset regression (BSR) (adjusted R-squared maximum as screening criteria) were utilized for predictor selection in training cohort, as described in the previous article (19). The subset of variables initially identified through these three distinct methodologies was then subjected to a multivariate Cox regression in conjunction with a gradual stepwise backward regression procedure, aimed at refining and enhancing our final model selection. The optimization process was guided by the Akaike information criterion (AIC), thus ensuring optimal model fit. Furthermore, we conducted an extensive evaluation using the time-dependent receiver operating characteristic (ROC) curve analysis to compare these three distinct screening approaches.

Ultimately, the identified predictive variables were utilized to construct a nomogram model founded upon the multivariate Cox regression method. The CS equation was incorporated into a nomogram to devise a novel CS-centered survival prediction model for real-time prognostic evaluation. Furthermore, this CS-based nomogram quantified all included variables as points and calculated risk score for each patient. Subsequently, a risk classification system was constructed based on the optimal threshold of risk scores among all patients to execute risk stratification for these patients. The Kaplan–Meier survival analysis employing a log-rank test was conducted to assess the disparity in OS amongst various risk cohorts.

Finally, the discriminability of the novel CS-based nomogram was assessed using various methods including the concordance index (C-index) and ROC curve with the area under the ROC curve (AUC) in both training and validation groups. Calibration curves were used to compare the predicted survival rates from the nomogram with the actual survival rates. Furthermore, the decision curve analysis (DCA) was carried out to appraise the clinical utility of the nomograms by quantifying the net advantages at different threshold probabilities.

The statistical evaluation of this research was executed in R language (version 4.1.0). P-values <0.05 were interpreted as statistically significant under the two-tailed test.

## Results

### Patient characteristics

We finally included 935 spinal PBL patients between 2000 and 2015 who conformed to the inclusion-exclusion criteria, ultimately allocated 7:3 into a training cohort (n=654) and validation cohort (n=281). In the whole cohort, a substantial majority was observed to be presenting an age surpassing 60 years (63.4%), predominantly representing a male gender (55.2%) and white race (86.3%). Regarding tumor features, the majority of PBL of the spine were found within the vertebral column (69.6%), exhibiting as diffuse large B-cell lymphoma (DLBCL) subtype (82.0%) and at an I/II stage (64.3%). In regard to treatment modality, it appeared that the predominant number of individuals received chemotherapy (74.4%) alongside radiotherapy (59.0%), whereas merely 28.0% underwent surgical resection. **Table 1** displays the baseline characteristic in detail.

**Table 1:** Clinicopathologic characteristics of lymphomas of the spine.

Characteristics	Total cohort (N=935)	Training group (N=654)	Validation group (N=281)	P value
Age at diagnosis				0.043
<40	111 (11.9%)	83 (12.7%)	28 (10.0%)	
40-60	231 (24.7%)	147 (22.5%)	84 (29.9%)	
≥60	593 (63.4%)	424 (64.8%)	169 (60.1%)	
Sex				0.256
Male	516 (55.2%)	353 (54.0%)	163 (58.0%)	
Female	419 (44.8%)	301 (46.0%)	118 (42.0%)	
Race				0.251
White	807 (86.3%)	570 (87.2%)	237 (84.3%)	
Non-white	128 (13.7%)	84 (12.8%)	44 (15.7%)	
Tumor site				0.715
Vertebral column	651 (69.6%)	453 (69.3%)	198 (70.5%)	
Sacrum/pelvic bone	284 (30.4%)	201 (30.7%)	83 (29.5%)	
Tumor histology				0.614
DLBCL	685 (73.3%)	476 (72.8%)	209 (74.4%)	
Non-DLBCL	250 (26.7%)	178 (27.2%)	72 (25.6%)	
Tumor stage				0.090
I/II	601 (64.3%)	409 (62.5%)	192 (68.3%)	
III/IV	334 (35.7%)	245 (37.5%)	89 (31.7%)	
Surgery				0.320
No	673 (72.0%)	477 (72.9%)	196 (69.8%)	
Yes	262 (28.0%)	177 (27.1%)	85 (30.2%)	
Radiotherapy				0.873
No	383 (41.0%)	269 (41.1%)	114 (40.6%)	
Yes	552 (59.0%)	385 (58.9%)	167 (59.4%)	
Chemotherapy				0.531
No	239 (25.6%)	171 (26.1%)	68 (24.2%)	
Yes	696 (74.4%)	483 (73.9%)	213 (75.8%)	
Rural-urban				0.540
Non-metropolitan	109 (11.7%)	79 (12.1%)	30 (10.7%)	
Metropolitan	826 (88.3%)	575 (87.9%)	251 (89.3%)	
Household income				0.383
<$70000	559 (59.8%)	397 (60.7%)	162 (57.7%)	
≥$70000	376 (40.2%)	257 (39.3%)	119 (42.3%)	
Marital status				0.612
Single	382 (40.9%)	261 (39.9%)	121 (43.1%)	
Married	522 (55.8%)	372 (56.9%)	150 (53.4%)	
Unknown	31 (3.3%)	21 (3.2%)	10 (3.6%)	

DLBCL, diffuse large B-cell lymphoma.

### Conditional survival analysis of PBL of the spine

**Figure 1:**
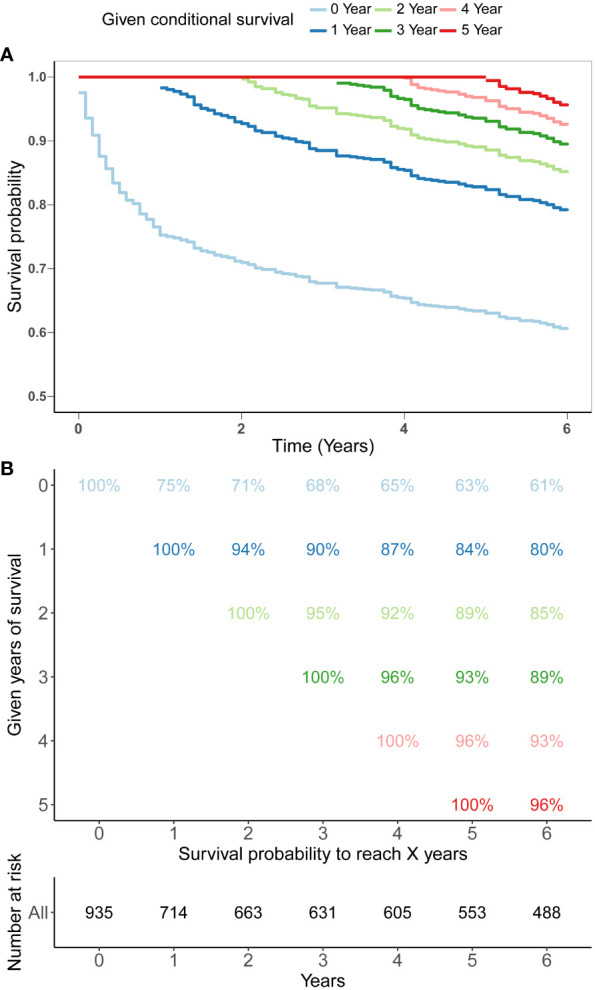
5-year conditional survival (CS) estimation after surviving 0-4 years in patients with PBL of the spine. Conditional survival curves **(A)** and their updated survival data adjusted for post-survival time **(B)**.

The SEER database provides a substantial sample size for this uncommon tumor, enabling reliable and robust survival analysis. Kaplan-Meier analysis was executed to determine the OS rate for these patients, revealing a survival rate of 68% and subsequently 63% at the 3-year and 5-year mark respectively (**Figures 1A, B**). We further investigated the CS patterning exhibited by these patients and discovered the survival of PBL in the spine progressively improved with time. (**Figures 1A, B**). The 5-year survival rate of patients was gradually progressed from 63% since diagnosis to 84% at the first conditional year, 89% at the second conditional year, 93% at the third conditional year and 96% at the fourth conditional year, indicating that the longer the patients have survived, the greater their likelihood of enduring for additional years.

### Development of a CS-based nomogram model

**Figure 2:**
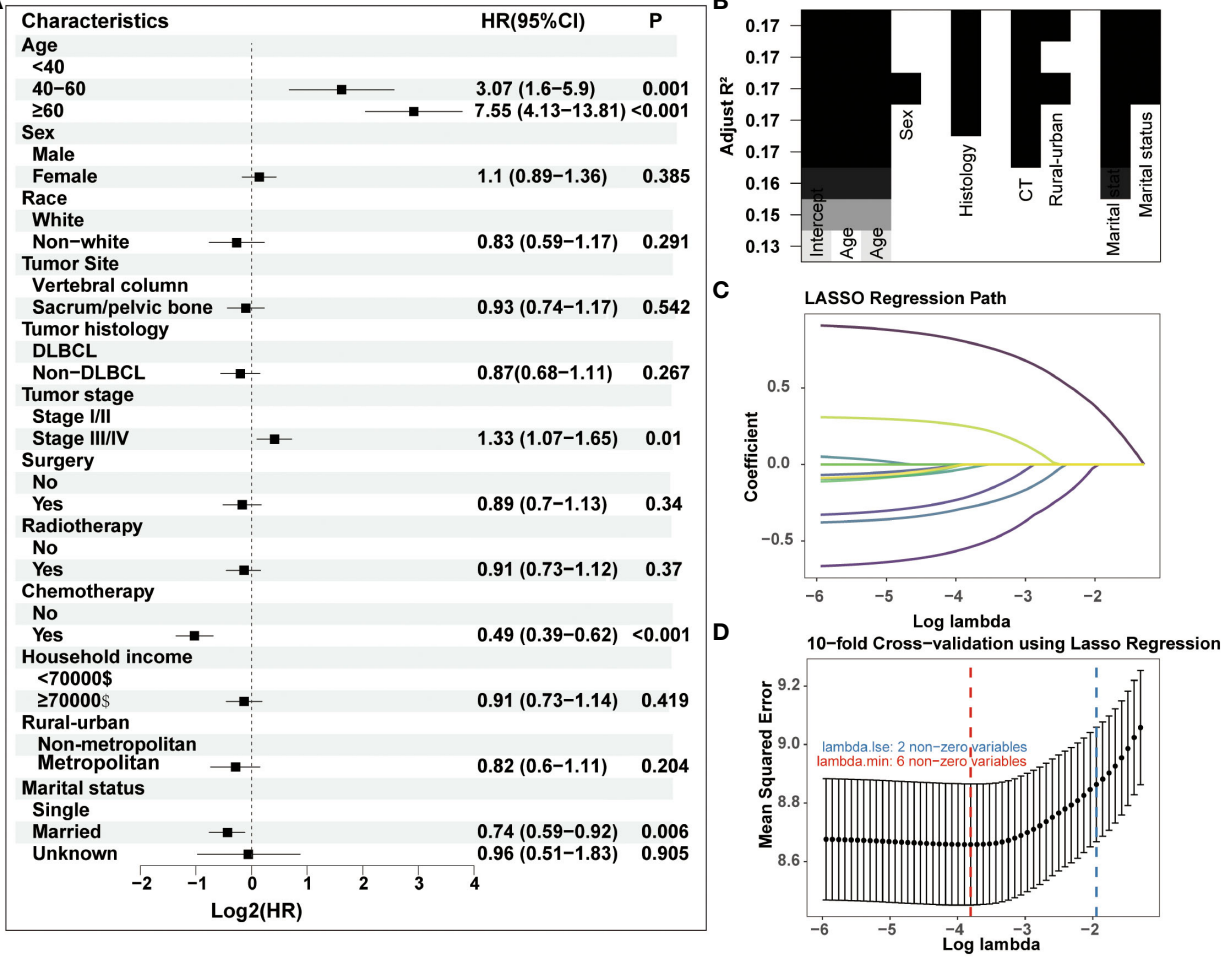
Three strategies including **(A)** univariate Cox regression, **(B)** BSR analysis and **(C–D)** LASSO regression were employed for prognostic factors identification. DLBCL, diffuse large B-cell lymphoma.

**Figure 3:**
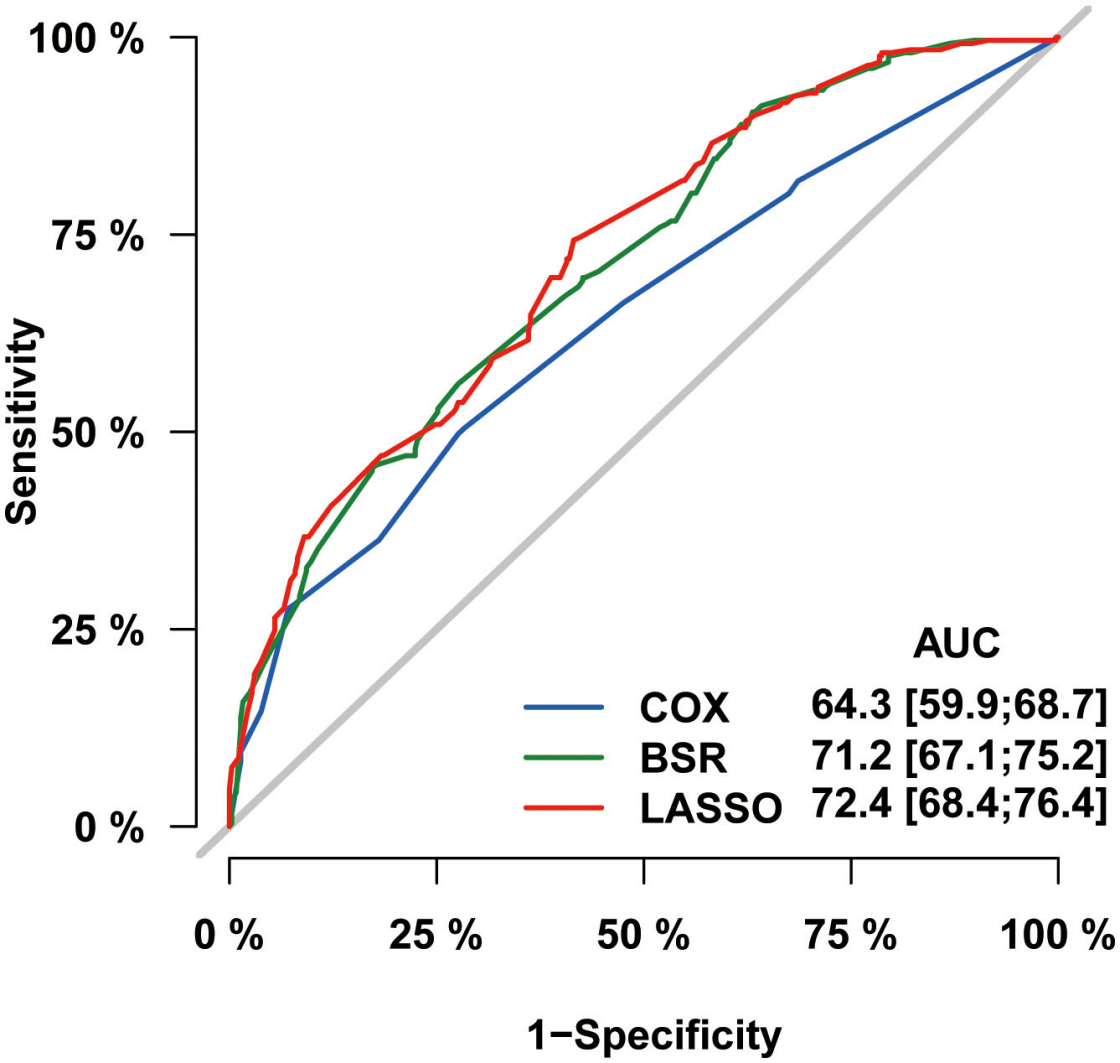
Comparison of three screening strategies for prognostic factors by ROC curve analysis.

**Figure 4:**
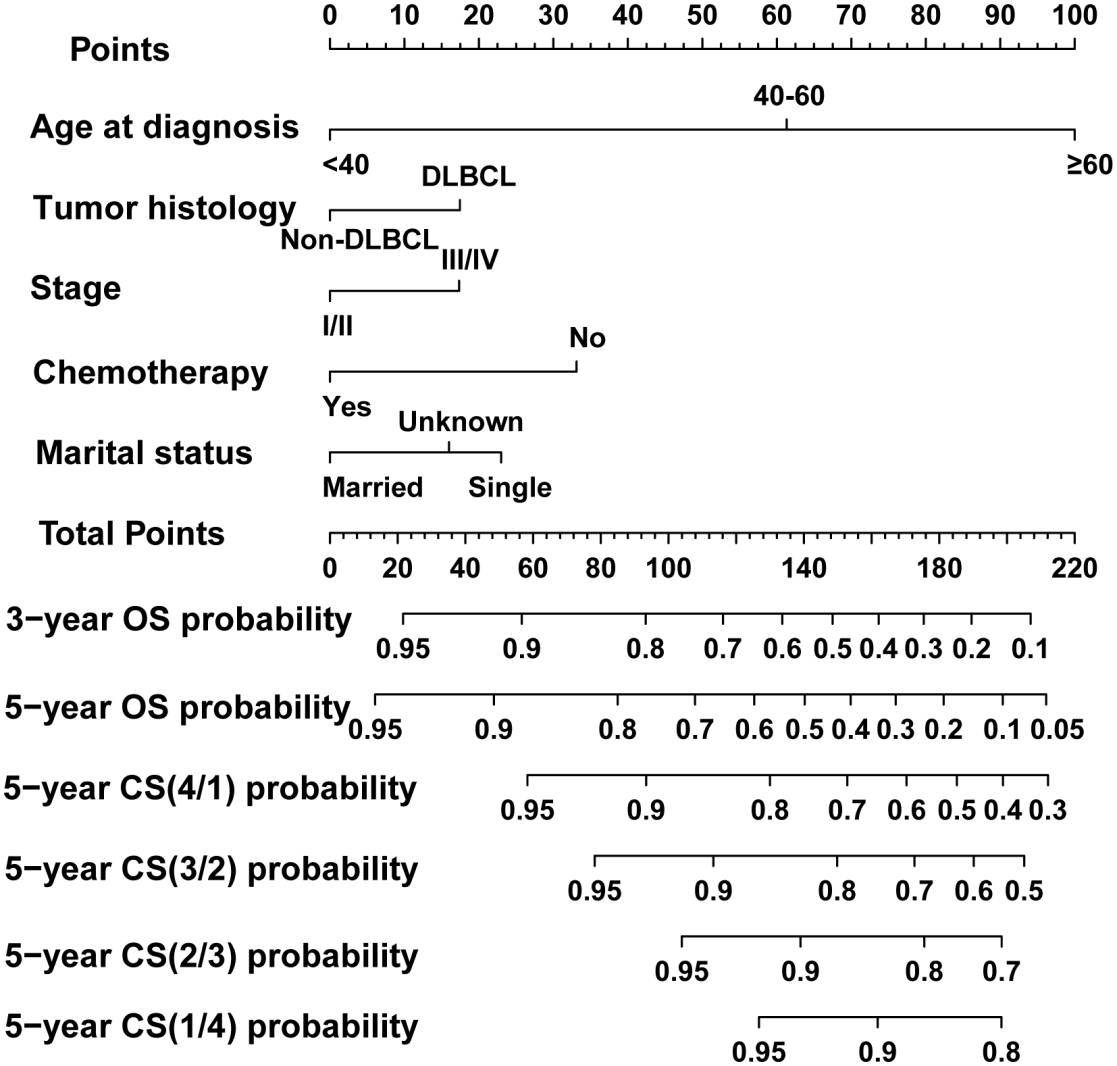
Dynamic conditional survival (CS)-based nomogram for predicting overall survival (OS) and CS for patients with PBL of the spine. DLBCL, diffuse large B-cell lymphoma.

In the process of prognostic factors identification, three strategies encompassing univariate Cox regression, LASSO regression and BSR analysis were employed (**Figure 2**). The forest plot exhibited that a collective of 4 (4/12) variables were identified grounded on univariate Cox analysis (P<0.05) (**Figure 2A**). A 6-element (6/12) combination was unearthed on the basis of the BSR analysis (**Figure 2B**). Furthermore, 6 (6/12) predictors were selected by LASSO regression under the condition of lambda.min (**Figures 2C, D**). Ultimately, subsequent revalidation through multivariate Cox stepwise backward regression identified 5 of the 6 variables which outlined by LASSO regression constituted the optimal model (AIC: Cox 4005.89; BSR 3913.87; LASSO 3906.28, see **Supplementary**). The ROC evaluation further confirmed this LASSO-selected subset exhibited superior discrimination with an AUC value of 72.4 (**Figure 3**). And the final selected predictors for survival prediction model were age, tumor histology, tumor stage, chemotherapy and marital status. Utilizing these variables and employing the CS formula, we successfully instigated a CS-based nomogram model for forecasting 3- and 5-year OS rates, along with 5-year CS rates of patients who have survived β years (**Figure 4**).

### Development of a risk stratification system

**Figure 5:**
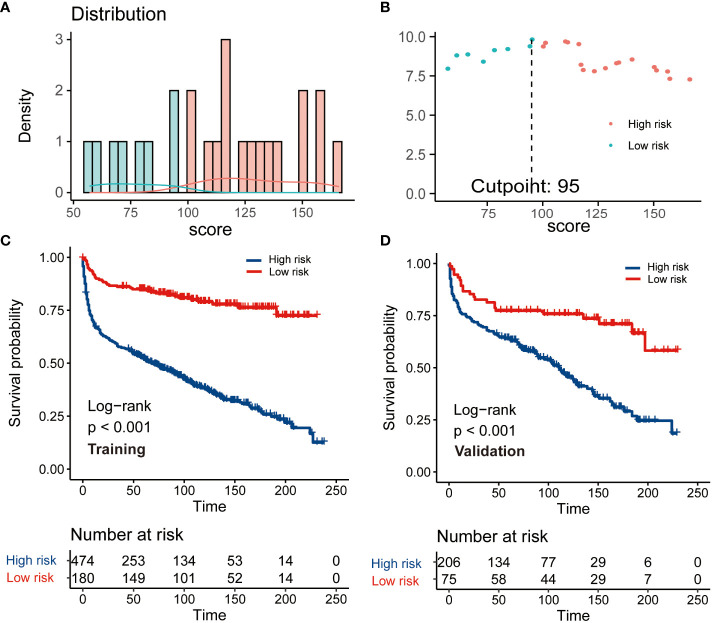
The construction and verification of a risk stratification system grounded on the condition survival nomogram. **(A)** Allocation of risk points; **(B)** The standardized log-rank statistics; **(C, D)** Kaplan-Meier analysis with log-rank test for diverse risk groups within both training and validation cohorts.

This CS-based nomogram model could quantify all included variables as points. Then, the risk score of each patient was calculated and the cut-off value was 95 according to R software for patient risk classification (**Figures 5A, B**). Based on the cut-off value, a risk stratification system for patients with PBL of the spine was successfully constructed based on the training cohort. The Kaplan–Meier analysis coupled with log-rank tests further elucidated that patients in elevated-risk groups exhibited notably inferior outcomes contrasted to those in reduced-risk groups within both the training and validation cohorts (**Figures 5C, D**), signifying that our risk categorization model exhibited strong discrimination.

### CS-based nomogram evaluation and validation

**Figure 6:**
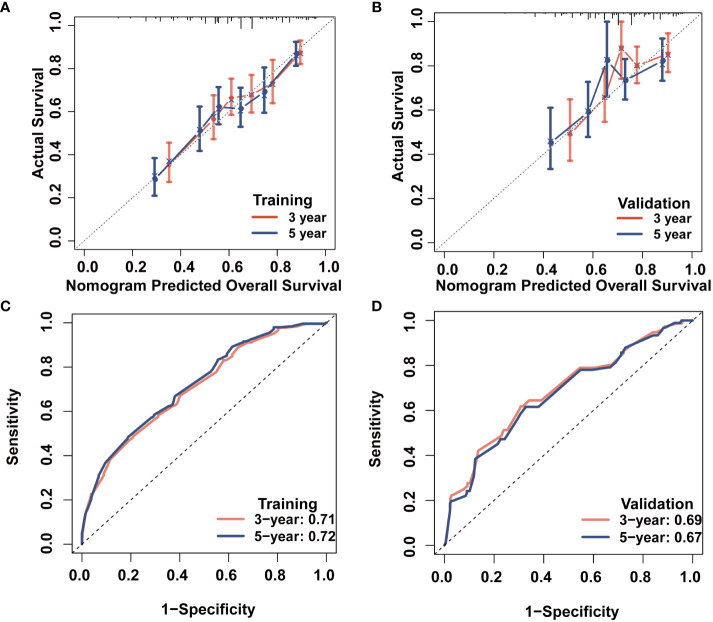
Survival prediction model evaluation. 3- and 5-year calibration plots of the training **(A)** and validation **(B)** cohort; 3- and 5-year time-dependent receiver operating characteristic (ROC) curves accompanied by area under curve (AUC) values in both training **(C)** and validation **(D)** cohorts.

**Figure 7:**
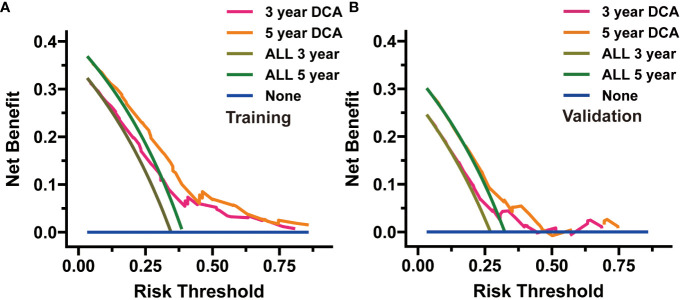
Decision curve analysis (DCA) was utilized to appraise the practicality of the condition survival (CS)-based nomogram in both the training **(A)** and validation **(B)** cohorts.

We evaluated the novel CS-based nomogram constructed utilizing the training cohort, with verification conducted using the validation cohort through various methods. The calibration curves of the training and validation cohorts were largely consistent with the diagonal, indicating that the projected probabilities correspond closely to the actual proportions (**Figures 6A, B**). The C-index values attained a value of 0.68 within the training cohort, while it was 0.67 within the validation group. The ROC analysis confirmed the nomogram’s satisfactory discriminatory capacity in both cohorts; the 3- and 5-year AUC values were 0.71 and 0.72 in the training group (**Figure 6C**), and the respective AUC values for the validation group were 0.69 and 0.67 for the same time periods (**Figure 6D**). The DCA curve demonstrates the net benefit gained from using the predictive model across various threshold probabilities. By analyzing the shape and trajectory of the DCA curve, clinicians can assess the clinical impact of using the predictive model at different threshold probabilities. A steep incline in the curve suggests substantial clinical gain, indicating that the model provides significant net benefit. The results of the DCA analysis for our model indicated a substantial net benefit and promising clinical prediction when patients incorporate the CS-nomogram as a tool for medical decision-making (**Figures 7A, B**).

## Discussion

While several articles have explored spinal intramedullary lymphomas (20), research specific to PBL of the spine remains substantially lacking. This is the first study to analyze the CS trend of spinal PBLs, and we observed that that the instantaneous survival rates of PBL of the spine progressively augmented over time. Meanwhile, through three different prognostic factor selection methods, we identified the best predicter subset for survival prediction model construction. Finally, we constructed and verified a unique CS-centered nomogram model for instantaneous and dynamic survival evaluation. Furthermore, based on this nomogram, we further designed a risk stratification system, thereby enhancing the efficacy of therapeutic strategies for these patients.

PBL typically portends a favorable prognosis (2, 10). Indeed, it is deemed to have the most favorable prognosis among all primary malignant bone neoplasms and better prognosis than secondary bone lymphoma (1, 21). And it is reported that tumors within the spinal column demonstrated substantially diminished survival compared to those in extremity bones, potentially due to nerve compression and the ensuing complications of this phenomenon (9), indicating that this subset of PBL requires further independent analysis. Instead of traditional survival analysis, we employed CS analysis to describe the survival pattern of spinal PBL and demonstrated that survival rate of PBLs of the spine progressively elevated over time with the 5-year survival probability evolving from 63% at the original diagnosis to 66.4%, 84%, 89%, 93% and 96% annually. This real-time feedback of survival and prognostic data will contribute to augmenting the survival reassurance of patients, mitigating psychological stress, and positively influencing the patients’ prognosis. In addition, we integrated CS into a nomogram model which considered the patient’s individualized clinicopathological factors to establish a novel CS-based prognosis prediction model with the ability of dynamic and personalized survival estimation.

Univariate Cox Regression, LASSO and BSR were selected as potential variable screening methodologies. These methods were selected to guarantee a comprehensive and robust examination, enabling us to address the complexity of our data effectively and build a model with more stable and excellent performance. Through a series of screening, our model finally utilized age, tumor histology, tumor stage, chemotherapy and marital status as predictive factors, demonstrating substantial correlation with prognosis. Advanced age and tumor stage represented significant and unfavorable prognostic indicators for survival among patients affected with PBL (2, 8, 11, 22, 23). Chemotherapy is the optimal therapeutic approach utilized for PBL patients, with rituximab in combination with anthracycline-based chemotherapy presently being endorsed (9, 22, 24). And our study also found that chemotherapy constituted the paramount factor influencing the outcomes. Moreover, the efficacy of surgical intervention in the management of PBL remains limited, and it usually applied for diagnostic purposes (2, 9, 24, 25). Despite the observation that our study failed to uncover a significant correlation between surgery and PBL prognosis, the therapeutic merit of surgical intervention warrants further exploration in relation to improving spinal PBL patient’s quality of life. Furthermore, the efficacy of radiotherapy remains contentious in the management of PBL patients. Some studies have indicated that radiotherapy may augment the local tumor control rate, thus potentially enhancing patient survival (9, 26); while some research reported that consolidation radiotherapy cannot improve PBL outcomes after receiving chemotherapy (22, 27, 28). A unanimous consensus has not yet been established for radiation treatment; hence, an intensified exploration of PBL subsets suitable for radiotherapy is indispensable. And our risk stratification system could aid in identifying individuals at elevated risk, facilitating exploration into potential applications of radiotherapy.

In the clinical practice, by inputting individual patient characteristics into our nomogram, clinicians can generate specific risk assessments that forecast outcomes with superior accuracy compared to traditional techniques. This precision permits a more refined understanding of each patient’s prognosis. Additionally, through our risk classification system, patients identified as high-risk may benefit from more aggressive therapies, whereas low-risk individuals might be exempted from superfluous treatments, thereby reducing potential adverse effects and optimizing resource utilization. And by providing a visual representation of risk, these tools can help demystify complex medical information, making it easier for patients to understand their prognosis and treatment options.

Our study was subjected to certain limitations. Firstly, this retrospective research was inherently biased. Secondly, the SEER database was devoid of imperative details, including the precise chemotherapy regimen, surgical procedure and radiotherapy dose, thereby potentially restricting our analysis. Thirdly, we failed to obtain sufficient cases for our model external validation. Fourth, while the study presents a novel CS-based nomogram for PBL of the spine, exploring the external validity of this model in different clinical settings could further validate its applicability. Future investigation could encompass multi-center studies to assess the performance of the nomogram within varying populations and healthcare systems. This method would aid in ascertaining any requisite modifications to assure its utility across various clinical contexts. And The progression of therapeutic modalities for PBL of the spine necessitates ongoing modifications to our model. Furthermore, employing more advanced machine learning methodologies can facilitate real-time modifications to the model predicated upon existing therapeutic protocols.

## Conclusion

In summary, based on the SEER database, this study estimated the CS trend of PBL of the spine and demonstrated that the real-time survival rate of PBL of the spine dynamically increased over time. We also successfully established and validated a novel CS-based prognosis prediction model with strong performance for survival estimation. Furthermore, a nomogram-based risk classification system was also designed for risk stratification for these patients, thereby aiding in optimization of clinical management of this disease. Future research demands further advancement in the formulation of predictive models with superior quality and performance for extensive applications, facilitating more meticulous therapeutic and follow-up strategies customization for patients afflicted with these rare tumor types.

## Data availability statement

The original contributions presented in the study are included in the article/**Supplementary Materials**, further inquiries can be directed to the corresponding author/s.

## Ethics statement

Ethical review and approval was not required for the study on human participants in accordance with the local legislation and institutional requirements. Written informed consent from the patients/participants or patients/participants’ legal guardian/next of kin was not required to participate in this study in accordance with the national legislation and the institutional requirements.

## Author contributions

GZ: Formal analysis, Methodology, Software, Supervision, Validation, Visualization, Writing – original draft, Writing – review & editing. ZY: Formal analysis, Validation, Writing – review & editing. HQ: Validation, Writing – review & editing. HH: Methodology, Writing – review & editing. ZG: Methodology, Supervision, Validation, Writing – review & editing.
