# Supplementary material for: Conditional survival of patients with primary bone lymphoma of the spine: how survival changes after initial diagnosis
Source: Front Oncol. 2024 May 1;14:1356947. doi: 10.3389/fonc.2024.1356947 (PMC11094224; doi:10.3389/fonc.2024.1356947)
Supplement: Supplementary file 1 [file DataSheet_1.docx]

**stepAIC(BSR, direction="backward")**

Start: AIC=3915.87

Surv(time, status == 1) ~ Age +Sex + Histology + Chemotherapy+ Rural +

Marital

Df AIC

- Rural 1 3913.9

<none> 3915.9

- Sex 1 3916.3

- Histology 1 3920.8

- Marital 2 3930.9

- Chemotherapy 1 3938.2

- Age 2 4011.2

Step: AIC=3913.87

Surv(time, status == 1) ~ Age + Sex + Histology + Chemotherapy+ Marital

Df AIC

<none> 3913.9

- Sex 1 3914.3

- Histology 1 3918.9

- Marital 2 3928.9

- Chemotherapy 1 3936.4

- Age 2 4009.7

Call:

coxph(formula = Surv(time, status == 1) ~ Age + Sex + Histology +

Chemotherapy+ Marital, data = train_data, x = T)

coef exp(coef) se(coef) z p

Age2 1.2593 3.5231 0.3349 3.760 0.00017

Age3 2.0974 8.1451 0.3125 6.712 1.92e-11

Sex2 -0.1787 0.8363 0.1147 -1.558 0.11920

Histology2 -0.3514 0.7037 0.1353 -2.597 0.00939

Chemotherapy2 -0.6450 0.5247 0.1260 -5.119 3.08e-07

Marital2 -0.5133 0.5985 0.1173 -4.375 1.21e-05

Marital3 -0.1217 0.8854 0.3280 -0.371 0.71059

Likelihood ratio test=151.6 on 7 df, p=< 2.2e-16

n= 654, number of events= 339

**> stepAIC(LASSO,direction="backward")**

Start: AIC=3907.22

Surv(time, status == 1) ~ Age + Histology + Stage + Radiotherapy + Chemotherapy+

Marital

Df AIC

- Radiotherapy 1 3906.3

<none> 3907.2

- Histology 1 3913.0

- Stage 1 3913.3

- Marital 2 3920.7

- Chemotherapy 1 3932.7

- Age 2 4000.1

Step: AIC=3906.28

Surv(time, status == 1) ~ Age + Histology + Stage + Chemotherapy+ Marital

Df AIC

<none> 3906.3

- Histology 1 3911.7

- Stage 1 3914.3

- Marital 2 3919.7

- Chemotherapy 1 3931.4

- Age 2 3998.3

Call:

coxph(formula = Surv(time, status == 1) ~ Age + Histology + Stage +

Chemotherapy+ Marital, data = train_data, x = T)

coef exp(coef) se(coef) z p

Age2 1.2585 3.5200 0.3349 3.757 0.000172

Age3 2.0524 7.7865 0.3112 6.595 4.24e-11

Histology2 -0.3581 0.6990 0.1342 -2.669 0.007617

Stage2 0.3560 1.4276 0.1112 3.203 0.001362

Chemotherapy2 -0.6796 0.5068 0.1261 -5.387 7.17e-08

Marital2 -0.4717 0.6239 0.1123 -4.200 2.67e-05

Marital3 -0.1438 0.8661 0.3279 -0.438 0.661055

Likelihood ratio test=159.2 on 7 df, p=< 2.2e-16

n= 654, number of events= 339

**> stepAIC(COX,direction="backward")**

Start: AIC=4005.89

Surv(time, status == 1) ~ Stage + Stage + Chemotherapy+ Marital

Df AIC

<none> 4005.9

- Marital 2 4010.0

- Stage 1 4015.3

- Chemotherapy 1 4043.3

Call:

coxph(formula = Surv(time, status == 1) ~ Stage + Stage + Chemotherapy+

Marital, data = train_data, x = T)

coef exp(coef) se(coef) z p

Stage2 0.3817 1.4647 0.1116 3.421 0.000623

Chemotherapy2 -0.7773 0.4597 0.1181 -6.584 4.58e-11

Marital2 -0.3189 0.7270 0.1112 -2.869 0.004124

Marital3 -0.1994 0.8193 0.3282 -0.607 0.543557

Likelihood ratio test=53.62 on 4 df, p=6.333e-11

n= 654, number of events= 339

>
